# Resurgence of SARS-CoV-2 Delta after Omicron variant superinfection in an immunocompromised pediatric patient

**DOI:** 10.1186/s12985-023-02186-w

**Published:** 2023-10-27

**Authors:** Arghavan Alisoltani, Lacy M. Simons, Maria Francesca Reyes Agnes, Taylor A. Heald-Sargent, William J. Muller, Larry K. Kociolek, Judd F. Hultquist, Ramon Lorenzo-Redondo, Egon A. Ozer

**Affiliations:** 1 Department of Medicine, Division of Infectious Diseases, Northwestern University Feinberg School of Medicine, Chicago, IL 60611 USA; 2 Center for Pathogen Genomics and Microbial Evolution, Northwestern University Havey Institute for Global Health, Chicago, IL 60611 USA; 3 Ann & Robert H. Lurie Children’s Hospital of Chicago, Chicago, IL 60611 USA

**Keywords:** SARS-CoV-2, Variants of concern, Delta, Omicron, Superinfection

## Abstract

**Background:**

Persistent SARS-CoV-2 infection in immunocompromised hosts is thought to contribute to viral evolution by facilitating long-term natural selection and viral recombination in cases of viral co-infection or superinfection. However, there are limited data on the longitudinal intra-host population dynamics of SARS-CoV-2 co-infection/superinfection, especially in pediatric populations. Here, we report a case of Delta-Omicron superinfection in a hospitalized, immunocompromised pediatric patient.

**Methods:**

We conducted Illumina whole genome sequencing (WGS) for longitudinal specimens to investigate intra-host dynamics of SARS-CoV-2 strains. Topoisomerase PCR cloning of Spike open-reading frame and Sanger sequencing of samples was performed for four specimens to validate the findings. Analysis of publicly available SARS-CoV-2 sequence data was performed to investigate the co-circulation and persistence of SARS-CoV-2 variants.

**Results:**

Results of WGS indicate the patient was initially infected with the SARS-CoV-2 Delta variant before developing a SARS-CoV-2 Omicron variant superinfection, which became predominant. Shortly thereafter, viral loads decreased below the level of detection before resurgence of the original Delta variant with no residual trace of Omicron. After 54 days of persistent infection, the patient tested negative for SARS-CoV-2 but ultimately succumbed to a COVID-19-related death. Despite protracted treatment with remdesivir, no antiviral resistance mutations emerged. These results indicate a unique case of persistent SARS-CoV-2 infection with the Delta variant interposed by a transient superinfection with the Omicron variant. Analysis of publicly available sequence data suggests the persistence and ongoing evolution of Delta subvariants despite the global predominance of Omicron, potentially indicative of continued transmission in an unknown population or niche.

**Conclusion:**

A better understanding of SARS-CoV-2 intra-host population dynamics, persistence, and evolution during co-infections and/or superinfections will be required to continue optimizing patient care and to better predict the emergence of new variants of concern.

**Supplementary Information:**

The online version contains supplementary material available at 10.1186/s12985-023-02186-w.

## Background

Since the declaration of the coronavirus disease 2019 (COVID-19) pandemic in March 2020, severe acute respiratory syndrome coronavirus 2 (SARS-CoV-2), the causative agent of the disease, has been subject to ongoing genetic diversification. This has resulted in the periodic emergence of new “variants of concern” (VOCs) with properties that confer enhanced fitness including heightened infectivity, transmissibility, and/or immune evasion [1–5]. To date, the named VOCs have included the Alpha (B.1.1.7*), Beta (B.1.351*) [1], Gamma (P.*) [3, 4], Delta (B.1.617.2, AY.*) [6, 7], and Omicron variants (B.1.1.529 and BA.*, BQ.*, BF*) [2, 5].

The Delta VOC emerged in India in late 2020 with six unique clade-defining mutations in the Spike open reading frame responsible for host cell attachment and entry (T19R, del156-157, R158G, L452R, P681R, D950N) [8]. Compared to prior variants, the Delta VOC was associated with higher viral loads, shorter incubation periods, increased transmissibility, and higher rates of reinfection [8, 9]. These fitness advantages enabled Delta to become globally dominant throughout the second half of 2021. After its emergence in September 2021, the Omicron variant rapidly displaced the Delta variant to become globally predominant by early 2022. The Omicron variant (and its sublineages) contain several Spike mutations that collectively confer increased transmissibility and shorter incubation periods compared to the Delta VOC [10–12]. Notably, this substantial genetic drift enabled widespread immune escape, including neutralizing antibodies elicited by vaccination and prior infection.

When two distinct SARS-CoV-2 variants are circulating at the same time, they may result in a co-infection (two simultaneous infections) or superinfection (two sequential infections) within a single host. Co-infections and superinfections with different variants can lead to more severe disease, complicate treatment strategies, and can potentiate the emergence of recombinant variants with unique phenotypic properties [13, 14]. Fortunately, co-infections and/or superinfections with distinct variants of SARS-CoV-2 have been rarely reported, with only a handful of studies documenting such cases [13–18]. In two separate studies conducted during the period of Delta and Omicron co-circulation, the prevalence of co-infection was estimated to be about 0.2% in the study populations [15, 17]. However, immunocompromised or immunosuppressed hosts who develop persistent infection with SARS-CoV-2 are more likely to develop viral superinfections, including with other SARS-CoV-2 variants [19–21]. Persistent infection in immunocompromised hosts has already been shown to result in the *de novo* emergence of immune resistance mutations and may be one source for the ongoing evolution of novel VOCs [22–24].

Here, we present a unique case of an immunocompromised pediatric patient with persistent infection with the SARS-CoV-2 Delta variant who experienced a transient superinfection with the Omicron variant before the resurgence of Delta.

## Methods

### Sample collection

As part of an established protocol at the Center for Pathogen Genomics and Microbial Evolution at Northwestern Feinberg School of Medicine, residual diagnostic specimens of individuals testing positive for SARS-CoV-2 at the Ann & Robert H. Lurie Children’s Hospital of Chicago were collected and stored (IRB 2020–3792). For this patient, all available residual clinical samples collected for COVID-19 testing between January and February 2022 were pulled for analysis.

### Viral load determination

The QIAamp Viral RNA Minikit (Qiagen) was used to extract viral RNA from nasopharyngeal specimens. Viral load determination was performed by quantitative reverse transcription and polymerase chain reaction (qRT-PCR), utilizing the CDC 2019-nCoV qRT-PCR Diagnostic Panel with N1 and RNase P probes as previously described [https://www.cdc.gov/coronavirus/2019-ncov/lab/rt-pcr-panel-primer-probes.html]. Specimen quality was assessed by cycle threshold (Ct) values for human RnaseP; specimens with an RNaseP Ct value greater than 35 were considered low quality and excluded from further analysis.

### Illumina WGS and data processing

Complementary DNA (cDNA) synthesis of viral RNA was carried out using the SuperScript IV First Strand Synthesis Kit (Thermo) with random hexamers in accordance with the manufacturer’s specifications. Amplification of the cDNA was performed utilizing two non-overlapping primer pools, which were created using Primal Scheme and provided by the Artic Network (version 4.1 release [https://www.protocols.io/view/ncov-2019-sequencing-protocol-bp2l6n26rgqe/v1]. Sequencing library preparation of genome amplicon pools was performed using the SeqWell plexWell 384 kit per manufacturer’s instructions. Pooled libraries were sequenced on the Illumina MiSeq using the V2 500 cycle kit. To process data and generate the consensus sequence based on the paired-end sequencing reads we used a previously-described analytical pipeline [25]. The PANGO (Pango v.4.2; data update: v1.18.1.1) tool was used to determine the lineage of each SARS-CoV-2 consensus sequence. We then use the consensus sequences to generate multiple sequence alignments using MAFFT (v7.475) [26]. Maximum Likelihood (ML) phylogeny was inferred with IQ-TREE (version 2.0.7) [27] using its Model Finder function [28] before each analysis to estimate the nucleotide substitution model best-fitted for each dataset by means of Bayesian information criterion (BIC). We assessed the tree topology for each phylogeny both with the Shimodaira–Hasegawa approximate likelihood-ratio test (SH-aLRT) [29] and with ultrafast bootstrap (UFboot) [30] with 1000 replicates each. We built additional ML phylogenies for these consensus sequences using a similar method while including all genomes publicly available in GISAID collected during the time of hospitalization from Chicago and Cook County (n = 1,345; Supplementary Table 1) using the SARS-CoV-2 reference genome Wuhan-Hu-1 (NC_045512) to root the tree.

To assess the distribution of circulating variants in the region at the time of this case, we retrieved 2,031 high-quality SARS-CoV-2 genomic sequences from isolates collected in Chicago and Cook County (n = 2,031) between January 2021 and March 2022 from the GISAID database [18]. We determined the clade frequency on each date, and we also provided the 7-day moving average of the clade relative frequency at each date (including the clade frequencies for the targeted date and 6 days before that date). The ggplot2 R package [31] was utilized to create visual representations depicting the changes in relative abundances of SARS-CoV-2 clades.

### TOPO PCR cloning of Spike open-reading frame

Primer pairs targeting genomic position 21,421 − 23,114 in the Wuhan reference genome (GenBank accession MN908947.3) were designed and utilized for RT-PCR of samples along the timeline of infection to confirm the presence of two unique viruses in these specimens. The 1,713 nucleotide (nt) amplicon covers the first 1,551 nt of SARS-CoV-2 Spike (genomic positions: 21,563 − 23,114). RT-PCR was conducted using the SSIV One-step RT-PCR Kit (Thermo Scientific, cat# 12,594,100) under the following parameters: reverse transcription at 45 °C for 20 min, inactivation at 98 °C for two minutes, 40 cycles of 98 °C for 10 s, 55 °C for 10 s, and 72 °C for 1 min, and lastly followed by a final extension at 72 °C for 10 min.

TOPO cloning was performed on selected specimens using Thermo CloneJET PCR Cloning Kit (Thermo Scientific, cat K1232) according to manufacturers’ instructions. Colonies were screened by restriction digest, gel electrophoresis, and subjected to Sanger sequencing. Sanger reads were processed using DNAStar’s Sanger/ABI Assembly to generate consensus sequences by using sequences obtained with both forward and reverse primers. Forward and reverse sequences overlapped in the middle of the fragment for approximately 100–400 base pairs. Nucleotides were trimmed from the ends of each sequence until the chromatograms were resolved. Consensus sequences were aligned using MAFFT (v7.475) [26] and the alignment was trimmed to remove leading or trailing nucleotides not shared by all sequences in the alignment for a total sequence length of 1600 nt. ML phylogeny was inferred from the trimmed alignment with IQ-TREE (version 2.0.7) [27].

### Determining the persistence of VOCs during the course of COVID-19 pandemic

To determine the persistence of VOCs during the COVID-19 pandemic, we retrieved 13,793,965 high-quality SARS-CoV-2 genomic data collected between December 2019 and February 2023 from the GISAID database (access date 05-15-2023) [32]. The ggplot2 R package [31] was utilized to create visual representations depicting the changes in relative and absolute abundances of SARS-CoV-2 VOCs. We then included all VOC/VOI genomes collected since June 2022 (n = 226) to visualize the phylogenetic tree for comparison with genomes collected during the peak of spread for each variant (n = 2,195). The month with the highest number of globally collected genomes was considered the peak of spread for each variant. In the initial phase of our sampling process, we narrowed down the entire GISAID dataset to include only the genome sequences collected during the peak month for each VOC/VOI, amounting to 1,182,641 sequences across 191 countries. To perform stratified random sampling on this subset, we utilized the “stratified” function from the “splitstackshape” library in R to divide the data into subgroups, in this case, countries, and then selected 15 random genome sequences from each country. We determined the first quartile of the total number of genome sequences for each country to be 14.8, and set this as the goal sample size (15). This ensured that there was a sufficient number of observations for random sampling within each country. If a country’s available observations were fewer than the desired sample size of 15, we included all of the available observations for that country in the final sample. This random sampling process aims to capture diversity and account for potential biases that may arise due to factors such as geographical distribution. ML phylogeny was constructed for all GISAID retrieved genomes (n = 2,421; Table S1) using the same methodology described earlier. The previously inferred ML phylogenetic tree was used to estimate a time-scaled phylogeny with TreeTime (v0.7.6) [33]. Additionally, we randomly selected 200 (to match the number to genome sequences of Delta collected after June 2022) Delta genome sequences collected around the peak of Delta VOC prevalence (July 2021). Bayesian phylogenetic analyses were conducted using the BEAST 2 software (version 2.7.5) [34]. BEAST priors were introduced with BEAUTI v2.7.5 including a strict molecular clock model with a lognormal distribution of the evolutionary rate, using a previously estimated evolutionary rate for this dataset (4 × 10 − 4) as the prior for the mean. We assumed a GTR substitution model and a Coalescence Bayesian Skyline with a parameter dimension of 10 for both population and group size to model the population size changes through time. Markov chain Monte Carlo (MCMC) runs of at least 200 million states with sampling every 5000 steps were computed [35]. The convergence of MCMC chains was monitored using Tracer v.1.7.2, ensuring that the effective sample size (ESS) values were > 100 for each parameter estimated with a chain burning of 20%. We summarized the posterior distribution of the inferred trees as maximum clade credibility (MCC) tree using TreeAnotator v2.7.1 Lineage-through-time (LTT) plot was constructed from the combined posterior distribution of sampled tree topologies by also using Tracer v.1.7.2 This plot depicts the most probable accumulation of lineages over time based on the temporal distribution of branches along the phylogeny. Additionally, pairwise genetic distances between sequences measured as p-distance using MEGAX software (v10.1.8) were computed to gauge the genetic divergence within each of our two sample groups and between them. We used a Wilcoxon rank-sum test followed by False Discovery Rate (FDR) adjustment of p-values using the Benjamini-Hochberg procedure accounting for multiple comparisons to compare the genetic diversity observed within the SARS-CoV-2 Delta variant genome sequences collected around the peak of prevalence (n = 200) with the genetic diversity within those collected after June 2022 (n = 200) as well as the pairwise genetic divergence between these two time points.

## Results

### Patient history

The patient was a school-aged child previously diagnosed with a neuroinflammatory condition initially treated with the IFN-γ blocking antibody emapalumab-Iszg and ongoing therapy with steroids and monthly etoposide. The patient had a history of varicella encephalitis and remained on suppressive valacyclovir therapy. In early January 2022, the child developed upper respiratory tract infection symptoms and was diagnosed with COVID-19 at an outside healthcare facility (symptom day 0). Over the next 72 h the patient developed fever, decreased fluid intake, and difficulty breathing and was given glucocorticoid replacement with hydrocortisone (25 mg/m^2^ for “stress dosing”) by mouth every 6 h as an outpatient.

**Fig. 1:**
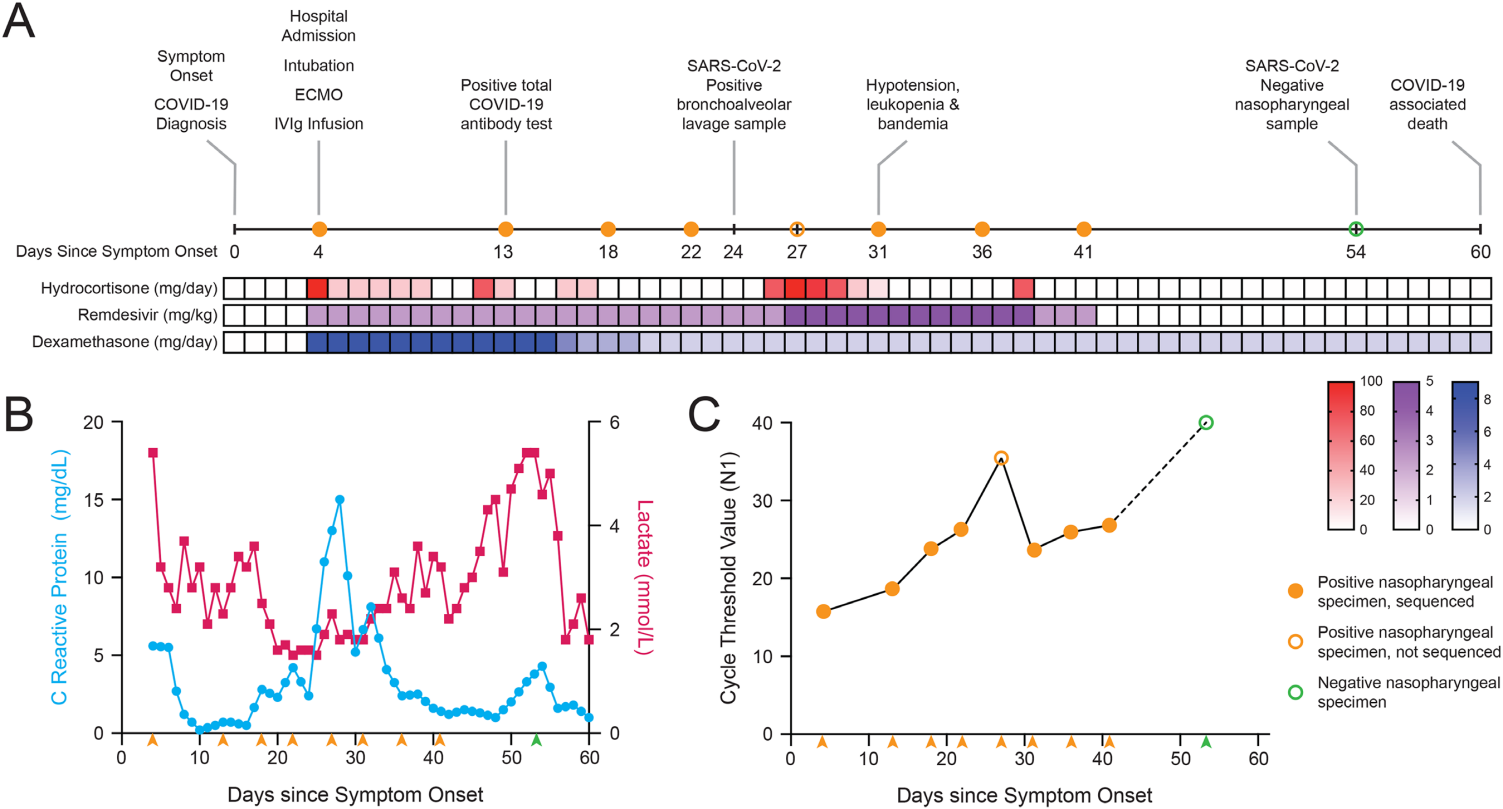
Case characteristics of an immunocompromised pediatric patient with persistent COVID-19. **(A)** Timeline of case patient’s illness, treatments, and COVID-19 testing. **(B)** Patient’s measured C-reactive protein (blue) and lactate levels (red) over the course of hospital admission. **(C)** SARS-CoV-2 quantitative RT-PCR cycle threshold (Ct) levels from available patient samples. X-axes for panels **B** and **C** are days since the onset of COVID-19 symptoms in the patient and arrowheads indicate SARS-CoV-2 testing dates

Four days after developing symptoms and first testing positive for SARS-CoV-2, the patient presented to the emergency department in respiratory distress. The patient was intubated and started on extracorporeal membrane oxygenation (ECMO) for respiratory support. A nasal swab collected on admission was positive for SARS-CoV-2 by a multiplexed PCR test, and the patient was started on remdesivir (5 mg/kg/day on day 1, then 2.5 mg/kg/day), hydrocortisone (100 mg on day 1, then 25 mg/day), and dexamethasone (9 mg/day) (Fig. 1A). The patient received an infusion of intravenous immune globulin (IVIg, 1 mg/kg/day) on days 5 and 6 following diagnosis and symptom onset for concern for COVID-19 myocarditis. At 11 days post-symptom onset, the patient experienced intermittent epistaxis and over the course of the next week developed hypotension, blood-streaked urine, dark tarry stools, pneumomediastinum, pneumoperitoneum, and elevated lactate levels (Fig. 1B). As the illness progressed, the patient had worsening oropharyngeal bleeding and epistaxis requiring nasal packing with oxymetazoline-soaked gauze. After 3 weeks, the patient developed hypotension, leukopenia, and bandemia. On day 31, the remdesivir dose was increased to 5 mg/kg/day and the hydrocortisone dose was increased to 100 mg/day followed by a 5-day taper. The patient subsequently failed an ECMO clamp trial (Fig. 1A).

Shortly thereafter, the patient had worsening pneumomediastinum and respiratory compliance with rising lactate levels. The ECMO circuit was changed with improved aeration of the lungs. Six weeks after onset, the patient’s lactate levels began rising again, and worsening bleeding from ECMO cannula site was noted. Nasal swabs had continued to be positive for SARS-CoV-2 by PCR every 4–6 days for almost the entire duration of his admission, but on day 51 the patient had his first negative PCR test. Unfortunately, his overall condition continued to deteriorate, and the patient died 60 days after his initial COVID-19 diagnosis (Fig. 1A).

### Population dynamics of SARS-CoV-2 virus during a prolonged infection

Quantitative RT-PCR revealed fluctuating SARS-CoV-2 viral loads in nasal swab specimens collected throughout the patient’s prolonged hospitalization (Fig. 1C). Cycle threshold (Ct) values were low on days 4 and 13 of illness, which indicated a high viral load at the onset of the infection. Viral Ct values increased in subsequent specimens up to a peak value of 36 on day 27, indicating a steady decrease in viral load. However, viral Ct values rebounded on day 31 and remained low through day 41. This rebound in viral load corresponded to a decline in the patient’s clinical status as indicated by worsening hypotension and increasing serum lactate levels (Fig. 1B). Viral load at day 51 was below the level of detection for the assay, suggesting clearance of the infection.

**Fig. 2:**
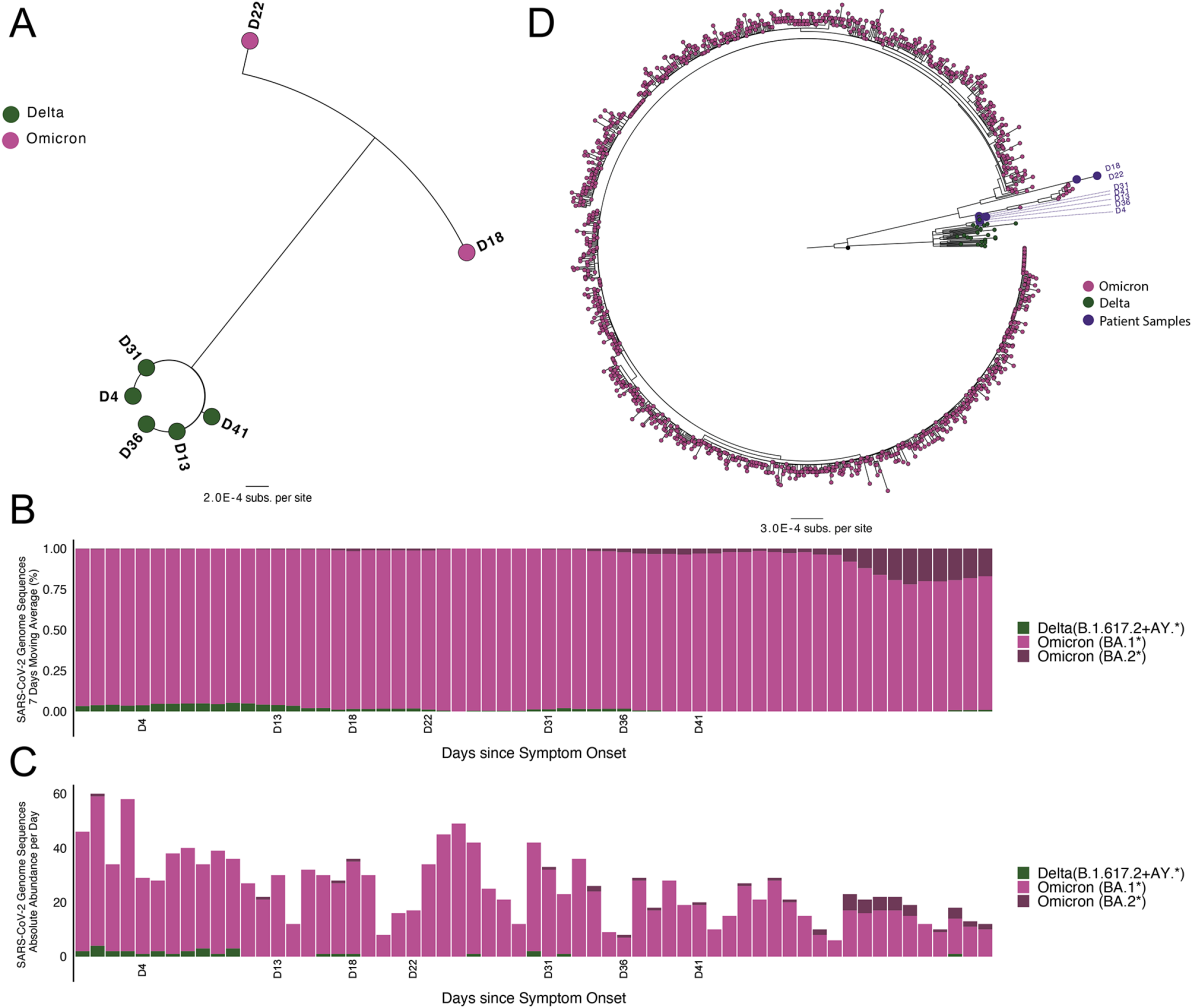
Sequencing of longitudinal nasopharyngeal isolates reveals a Delta-Omicron superinfection. **(A)** Maximum Likelihood phylogeny of the consensus SARS-CoV-2 whole genome sequences from Illumina short read sequencing. **(B)** Temporal dynamics of SARS-CoV-2 lineages (Relative abundance for seven days moving average) from Chicago and Cook County during the course of hospitalization. **(C)** Temporal dynamics of SARS-CoV-2 lineages (Absolute abundance per day) from Chicago and Cook County during the course of hospitalization. **(D)** Maximum likelihood phylogenetic tree of SARS-CoV-2 genome sequences from the patient samples (large purple dots) with all publicly available sequences from Chicago and surrounding Cook County collected between January and February 15, 2022 (black dots; n = 1,345) and deposited in the GISAID database

We performed whole-genome sequencing of SARS-CoV-2 from seven residual diagnostic specimens with sufficient viral load for sequencing (Ct values < 30), corresponding to days 4, 13, 18, 22, 31, 36, and 41 post-symptom onset. Phylogenetic analysis of the consensus sequences clustered the specimens into two groups (Fig. 2A). Specimens collected on days 4, 13, 31, 36, and 41 all belonged to Pango lineage AY.118, a Delta lineage, whereas specimens collected on days 18 and 22 were both identified as Pango lineage BA.1, an Omicron lineage. To confirm these results, independent nucleic acid extractions, library preparations, and sequencing runs were performed from separate aliquots of five specimens (Table S1), yielding identical lineage assignments. The consensus sequences obtained from independent runs align well, with most instances showing near-identical matches. However, in three specific samples (D18, D22, and D31) very minor variations (often one or two substitutions) were observed between the consensus sequences from the two runs (Table S1). All consensus sequences of Delta genomes (those runs we used in the study and deposited to GISAID) were found to be identical. The only differences detected between the consensus sequence of Day 41 having two additional nucleotide substitutions, C19955T and A20055G, with the first resulting in a missense mutation (nsp15 T112I). Among the omicron consensus sequences, the sequence from D18 included four additional nucleotide substitutions [C27807T, G28881A, G28882A, and G28883C (N:RG203KR)] compared to the D22 consensus sequence. While D22 included three additional nucleotide substitutions [C27874T (ORF7b:T40I), G28202A, G28881T (N:R203M)] compared to the D18 consensus sequence. No known remdesivir resistance-associated mutations were present in any isolate sequence. To confirm contemporaneous co-circulation of both these Delta and Omicron lineages in the region, we assessed the daily distribution of the most common lineages from publicly available sequences in the GISAID database from Chicago and surrounding Cook County over the dates of the patient’s infection (Fig. 2B and Fig. 2C). At the time of symptom onset, the proportion of new COVID-19 infections in Cook County caused by the Delta VOC was approximately 3–4%, which decreased to less than 1% by day 31 when Delta resurged in the patient (Fig. 2B). By day 18, when Omicron BA.1 was first detected in the patient, Omicron BA.1* lineages were causing greater than 98% of new COVID-19 infections in Cook County. Phylogenetic analysis of the consensus sequences with publicly available sequences in GISAID of isolates collected in Chicago and Cook County during the same time period confirmed that the patient isolates from days 4, 13, 31, 36, and 41 clustered with other contemporaneous Delta lineage sequences whereas the day 18 and 22 isolates clustered with other contemporaneous Omicron lineage sequences (Fig. 2D).

**Fig. 3:**
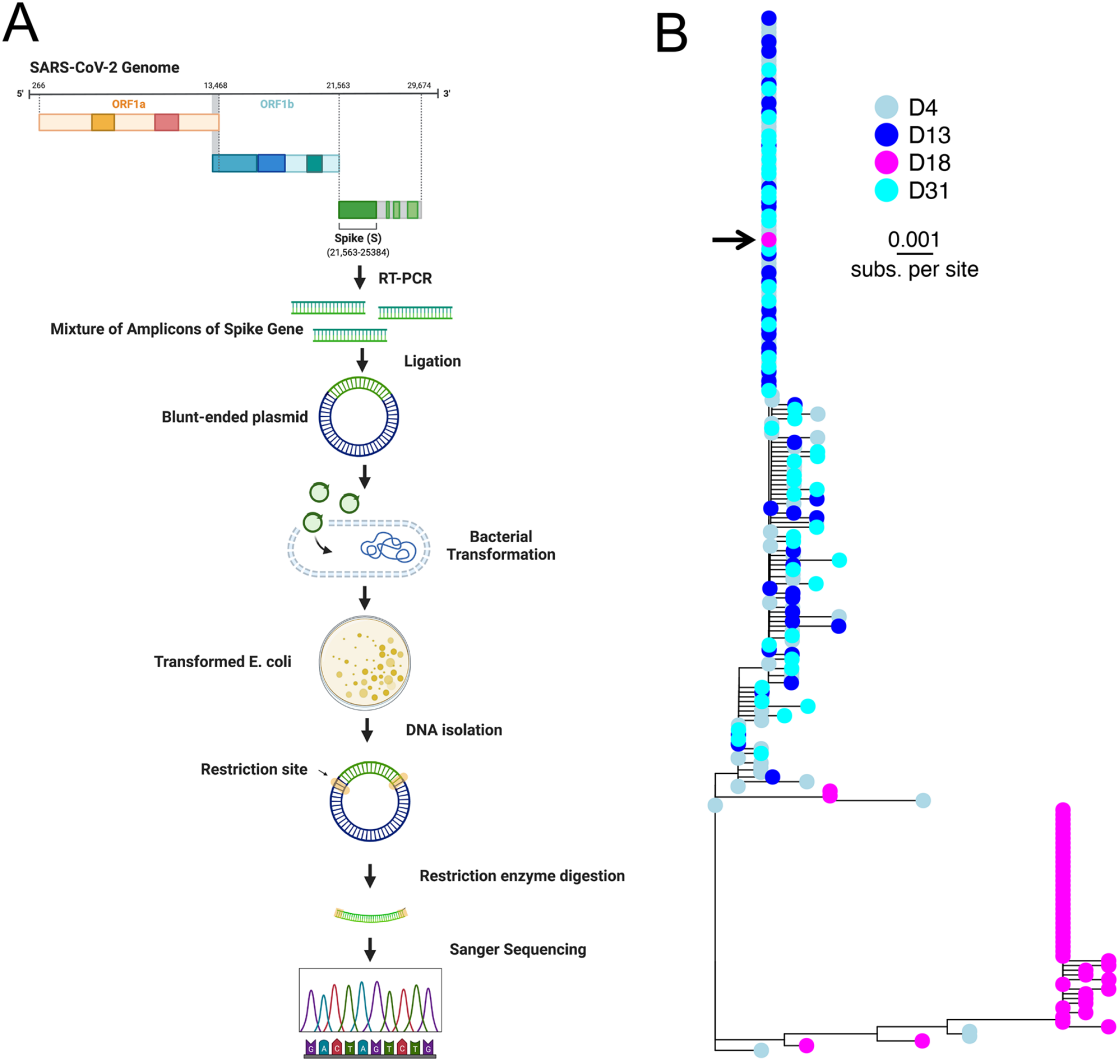
Single amplicon sequencing confirms Delta variant infection with a transient Omicron superinfection. **(A)** Workflow for Spike amplification, topoisomerase (TOPO) based cloning, and Sanger sequencing of specimens. Created with https://BioRender.com. **(B)** Quasispecies haplotypes of SARS-CoV-2 Spike region in four selected nasopharyngeal specimens. A maximum likelihood phylogenetic tree of the cloned SARS-CoV-2 Spike fragment sequences was generated from specimens collected on days 4 (light blue; n = 109), 13 (blue; n = 56), 18 (magenta; n = 52), and 31 (cyan; n = 58) since symptom onset. Arrow indicates Delta S gene clone sequence found in the otherwise Omicron-abundant day 18

The above deep sequencing results suggested that the patient was initially infected with the SARS-CoV-2 Delta variant followed by a transient superinfection with the Omicron variant. However, these results were based on short-read deep sequencing, which does not capture mutational linkage and may not be reliable for capturing low-frequency variants due to the inherent error rate. Therefore, to better understand when the Omicron superinfection started and if any recombinants arose intra-host, we performed Sanger sequencing of individual 1.5 kilobase (kb) Spike amplicons from 4 specimens at days 4, 13, 18, and 31 (Fig. 3A). 60 individual clones were sequenced for each timepoint plus an additional 60 clones from day 4. Sequences that did not span the entire amplicon or that yielded conflicting forward and reverse sequencing results were discarded (total of 109, 56, 52, and 58 sequences from days 4, 13, 18, and 31, respectively).

Phylogenetic analysis of the individual Spike amplicons revealed close clustering of sequences from days 4, 13, and 31 (Delta variant sequences) and a distinct clustering of Spike amplicons from day 18 (Omicron variant sequences) (Fig. 3B). All amplicons from days 4, 13, and 31 were consistent with a Delta variant infection, while nearly all amplicons from day 18 were consistent with an Omicron variant infection with the exception of a single Delta amplicon. These data suggest that the Omicron superinfection started sometime after day 13 with both variants present at day 18 prior to clearance of the Omicron superinfection by day 31. Notably, Ct values steadily increased over the first 22 days of infection, signifying that the superinfection was not associated with an increase in viral load. Shortly thereafter, a very high Ct value specimen at day 27 preluded the resurgence of the Delta variant at day 31, suggesting that the patient may have been close to clearing the infection in the nasopharynx before re-establishment or reseeding of the viral population by Delta.

### Persistent infections with Delta variant strains in the Omicron era

**Fig. 4:**
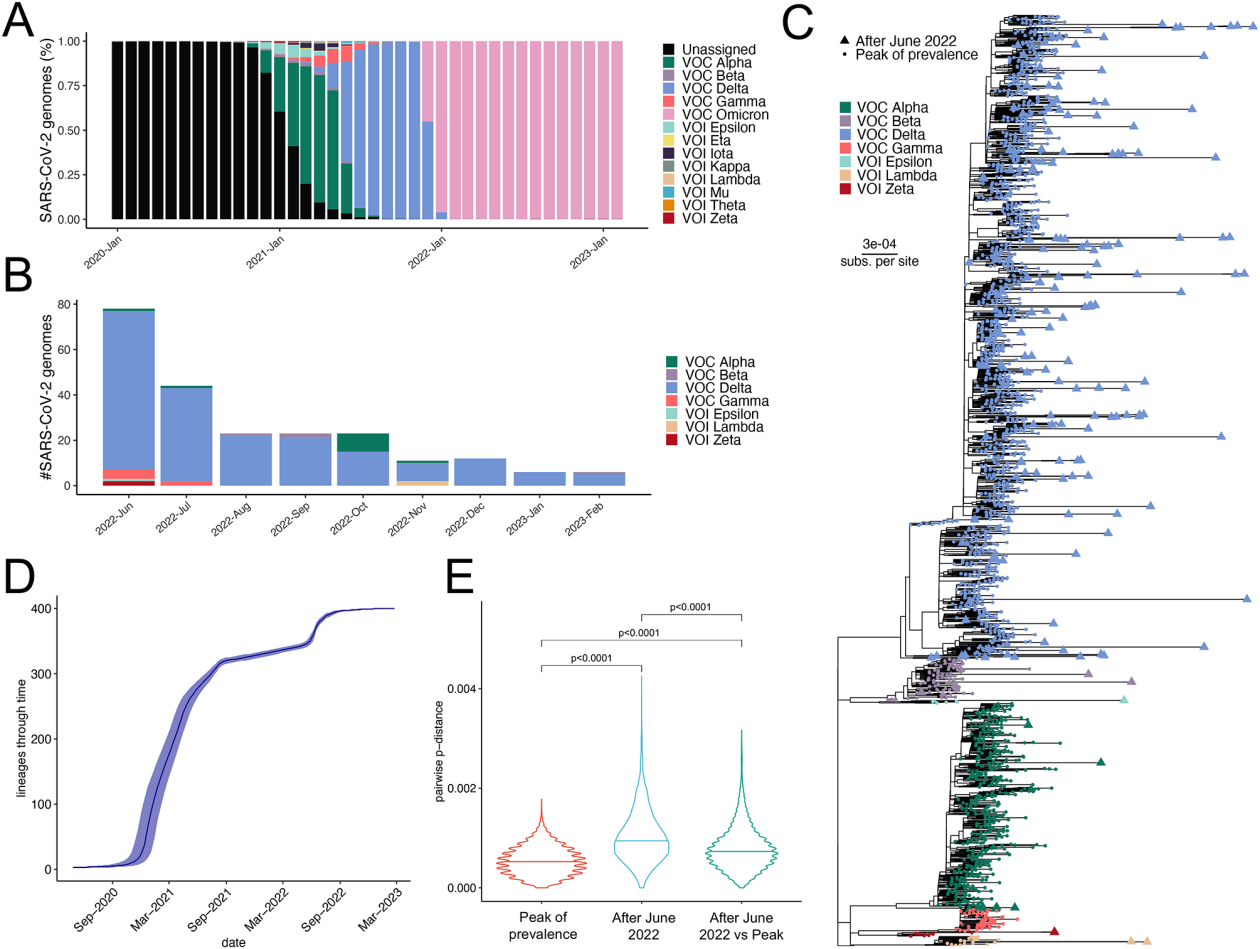
Persistence of Delta variants in public sequence data after the emergence of Omicron. **A)** Temporal dynamics of VOCs during the course of the pandemic based on 13,342,630 high-quality SARS-CoV-2 whole genome sequences from the GISAID database collected between December 2019 and February 2023. **B)** Temporal dynamics of non-Omicron SARS-CoV-2 genome sequences collected after June 2022. **C)** Maximum likelihood phylogenetic tree of non-Omicron variants collected after June 2022 (triangles; n = 226) and random subsamples of the same variants collected around their respective peak prevalence (circles; n = 2,195). **D)** Lineages-Through-Time (LTT) plot derived from Bayesian analysis of SARS-CoV-2 Delta variant genome sequences collected around the peak of prevalence (n = 200) and after June 2022 (n = 200). The plot chronologically represents the accumulation of lineages from September 2020 to March 2023. **E**) Violin plots displaying pairwise p-distance distributions of SARS-CoV-2 Delta variant genome sequences. Distributions represent all pairwise comparisons between genomes from the peak of prevalence (n = 200), post-June 2022 (n = 200), and the pairwise divergence between these two time points. Each “violin” width is indicative of the kernel density estimation of the data, presenting an overview of genetic divergence within and between the two sample groups. Thick line in each violin shows median pairwise p-distance of each group and whiskers indicate the upper and lower 1.5 interquartile ranges (IQR). P-values were calculated using the Wilcoxon rank-sum test and subjected to adjustment for False Discovery Rate (FDR) using the Benjamini-Hochberg procedure, accounting for multiple comparisons

Unlike most reported cases of superinfection where one variant outcompetes and supplants another, here we identified a unique case of a Delta variant infection with a transient Omicron variant superinfection. This suggests that Delta may have occupied an anatomic niche that enabled its longer-term persistence in an infected host compared to Omicron. Indeed, while the Omicron variant has been the globally predominant source of new infections since January of 2022 (Fig. 4A), new Delta lineage isolates have been reported as recently as February 2023. To explore this more thoroughly, we examined all publicly-deposited sequences in the GISAID database that were assigned a non-Omicron lineage after May 2022. This revealed a diminishing, but ongoing recovery of Delta lineage isolates through February of 2023 as well as the sporadic recovery of even earlier VOCs, such as Alpha and Gamma (Fig. 4B and Figure S1).

Phylogenetic analysis of VOC/VOI genomes collected after May 2022 confirmed the reported lineage designations in the database and reaffirmed the continued presence of the Delta variant through February 2023 (Fig. 4C and Figure S2). Additionally, this analysis revealed that the newer Delta isolates are genetically divergent from the earlier Delta isolates, suggesting that these specimens are representative of ongoing replication and evolution and are not simply misannotated (Fig. 4C**)**. To further assess genomic diversity among pre- and post-Omicron era Delta sequences, we randomly selected 200 Delta isolate sequences from the GISAID database collected around the time of peak Delta prevalence (July 2021) for comparison with the 200 Delta isolates collected between June 2022 and February 2023. Phylodynamics analysis of the SARS-CoV-2 Delta genome sequences collected around the peak of prevalence (n = 200) and after June 2022 (n = 200) reveals a continuing spread and diversification of this clade. The lineages-through-time plot (Fig. 4D) showcases a marked increase in lineage numbers from early 2021, signifying the rapid dominance of the Delta variant. This is consistent with records that indicate the Delta variant was first identified in late 2020 in India, but it took some time before it was recognized globally. However, post-January 2022, the deceleration in lineage accumulation suggests the waning prevalence of Delta, potentially due to competition with the Omicron variant. Despite the relative stasis in recent months, the sustained presence of Delta lineages after June 2022 underlines its continued circulation and divergence. Furthermore, our analysis of the pairwise p-distances confirms the observation from ML phylogeny (Fig. 4E). During the peak of Delta prevalence, we observed minimal genetic divergence among the samples. However, the samples collected after June 2022 exhibit a marked increase in genetic diversity, as demonstrated by the significantly higher within group pairwise p-distances, as well as an increase of genetic divergence when comparing the initial and more recent Delta sequences (Fig. 4E). This genetic diversification between the peak and post-June samples substantiates the Delta variant’s continuous evolution, even amidst the rise of Omicron lineages. These results suggest that the SARS-CoV-2 Delta variant continued to cause sporadic infections into 2023 despite the near complete global sweep of the Omicron variant. This may indicate that the Delta lineage has adaptive advantages that enable more persistent infections, perhaps in selected niches or patient populations.

## Discussion

We report a case of an immunocompromised pediatric patient infected with the SARS-CoV-2 Delta VOC, who experienced a transient superinfection with the Omicron VOC. Superinfection with two distinct strains of SARS-CoV-2 is rarely reported with an estimated prevalence of less than 0.2%. Nevertheless, these rare occurrences of co-infection and/or superinfection provide important opportunities for genetic recombination between distinct strains, which may lead to the emergence of novel variants. For example, several instances of intra-host recombination between the Delta and Omicron VOCs have been reported following co-infection or superinfection [15, 17]. Recombination events have been the source of several recombinant variants that have occasionally risen to global predominance, such as the Omicron recombinant lineage XBB [36].

The case reported here is unique in that the superinfection is transient, with later resurgence of the original variant. Most case reports of superinfection have found either displacement of the original variant or long-term persistence of both variants over time [13, 18–20]. To confirm these results, all deep sequencing was performed in at least two independent replicates from independent specimen aliquots with confirmation by Sanger sequencing of individual amplicons, all of which were consistent with this progression and verified the presence of both variants in at least one specimen (day 18). These controls make it unlikely that these results were due to error, contamination, or experimental artifacts.

The resurgence of the Delta variant in this patient was preceded by a drop in viral load, which may suggest the patient was close to clearing the infection in the nasopharynx prior to reseeding the infection by Delta. Phylogenetic analysis of the specimens found that the resurgent Delta isolates were nearly identical to the ones at initial infection while community incidence of Delta at the time of resurgence was less than 1%, suggesting that Delta infection persisted intra-host at a low level throughout the Omicron superinfection, perhaps in another anatomical compartment.

Despite the prolonged administration of remdesivir in this patient, no genetic changes undermining the therapeutic potential of remdesivir were detected, including any novel mutations in the nsp12 gene, that could explain either the persistence or resurgence of the Delta lineage virus or the Omicron superinfection. Although some studies reported de novo emergence of remdesivir resistance mutations [37, 38] the occurrences of such mutations in the GISAID repository continue to be consistently low, indicating their limited presence and lack of fitness [39]. The explanation for persistent viral recovery in this patient while on antiviral therapy remains unclear, though persistent or recurrent viral replication with remdesivir despite the absence or resistance mutations have been reported [40] and may result from a variety of factors including reduced immune clearance of virus or potential compromised penetration of remdesivir into the lung [41, 42].

Infection with the Omicron variant has been associated with an altered symptom profile, less severe disease, and better patient outcomes compared to the Delta variant [10–12, 43, 44]. On the contrary, the Delta variant has been associated with increased disease severity, higher viral loads, and a longer duration of positive PCR results compared to other variants [10, 45, 46]. This may be due to the greater reliance of Omicron Spike on the endosomal route of cell entry as opposed to TMPRSS2-mediated entry at the cell surface, which may alter viral tropism towards the upper as opposed to the lower respiratory tract [47, 48]. Indeed, it has been demonstrated that SARS-CoV-2 exhibits genetic variability with distinct quasispecies populations in the upper and lower respiratory tracts [49]. Furthermore, genetic compartmentalization of the virus between the oral cavity and nasopharynx has been described [50]. Taken together, these findings support intra-host viral niche adaptation and specificity that might provide opportunities for persistence and re-emergence of a particular variant in a host. Nevertheless, there is a scarcity of studies that comprehensively compare the dynamics of tissue-specific viral infections between the Delta and Omicron variants, especially in the context of a superinfection.

Besides the overall drop in viral load prior to Delta resurgence in our patient, there were also medical management changes immediately prior, including increased doses of remdesivir and hydrocortisone. As an alternate hypothesis, it is possible that treatment may alter the selective pressures and relative fitness of different SARS-CoV-2 variants during superinfection. For example, hydrocortisone has been shown to increase TMPRSS2 expression in vivo [51]. Given the preference of the Delta Spike protein for the TMPRSS2-mediated entry pathway, a change in hydrocortisone dosage could potentially provide a fitness advantage to Delta over Omicron viruses in the context of an already immunocompromised host. Nevertheless, this correlation remains hypothetical, and more research will be required to elucidate the impact of treatments in the context of superinfection with distinct viral variants.

The persistence of Delta in this patient over the course of a transient Omicron superinfection led us to explore whether there might be any evidence of Delta persistence in broader surveillance data. Despite the global population sweep of the Omicron variant in early 2022, our investigation of SARS-CoV-2 genomes collected after June 2022 yielded evidence of continued Delta transmission and evolution into 2023, albeit at low levels. Given the observed diversification of the newer isolates, these are unlikely to be due to metadata annotation errors alone. On one hand, these may be reflective of persistent or long-term infections in immunocompromised hosts and not of *de novo* transmission events. Alternately, it could be reflective of ongoing transmission in communities with relative social and/or geographic isolation or in under-sampled animal reservoirs that may have potential for occasional spillover into humans [52, 53]. Understanding the source of these rare infections is critical for genomic surveillance efforts aimed at monitoring potential emerging variants that may have a selective advantage as the global population gains broader immune protection to Omicron and its descendant sublineages.

## Conclusions

In summary, this study reports a unique case of an immunocompromised pediatric patient who experienced a persistent infection with SARS-CoV-2 Delta and a transient superinfection with SARS-CoV-2 Omicron. The data suggests that the Delta VOC may populate a unique niche within the host that enables its persistence even in response to subsequent superinfections and therapeutic changes. A better understanding of the anatomical population dynamics that occur during co-infection and superinfection is essential for optimizing treatment course and minimizing the risk of viral recombination. Furthermore, continued surveillance and research are necessary to monitor the dynamics of different variants and their persistence as the pandemic progresses. Evidence of long-term community transmission of the Delta variant after the emergence and predominance of Omicron suggests risks for variant re-emergence as the population gains lineage-specific immunity. Ultimately, understanding these factors will be crucial for effective public health measures, such as monitoring and treatments in susceptible populations, developing targeted therapeutics and vaccines, as well as anticipating future emerging and re-emerging infections.

### Electronic supplementary material

Below is the link to the electronic supplementary material.

Supplementary Material 1

Supplementary Material 2

## Data Availability

All consensus sequences generated in this study were deposited to GISAID (https://gisaid.org/). List of GISAID identifiers, along with information about the submitters, used in this study is provided in Supporting Information Table S1.

## Abbreviations

COVID-19: Coronavirus disease 2019
Ct: Cycle threshold
ECMO: Extracorporeal membrane oxygenation
GISAID: Global initiative on sharing all influenza data
IQR: Interquartile ranges
Kb: Kilobase
ML: Maximum likelihood
qRT-PCR: Quantitative reverse transcription and polymerase chain reaction
SARS-CoV-2: Severe acute respiratory syndrome coronavirus 2
SH-aLRT: Shimodaira–Hasegawa approximate likelihood-ratio test
TMPRSS2: Transmembrane serine protease 2
UFboot: Ultrafast bootstrap
VOC: Variant of concern
VOI: Variant of interest
WGS: Whole genome sequencing
