# Supplementary material for: Resurgence of SARS-CoV-2 Delta after Omicron variant superinfection in an immunocompromised pediatric patient
Source: Virol J. 2023 Oct 27;20:246. doi: 10.1186/s12985-023-02186-w (PMC10604949; doi:10.1186/s12985-023-02186-w)
Supplement: Supplementary file 1 — Supplementary Material 1 [file 12985_2023_2186_MOESM1_ESM.docx]

**Supporting Information Figure S1. Persistence of SARS-CoV-2 VOCs and VOIs following peak incidence.** Monthly incidences of SARS-CoV-2 VOCs/VOIs after reaching their respective peaks of prevalence during the course of the pandemic based on 13,342,630 high-quality SARS-CoV-2 whole genome sequenced data collected between December 2019 and February 2023 from the GISAID.

**Figure S2. Persistence of Delta variants in public sequence data after the emergence of Omicron.** Temporal dynamics of non-Omicron SARS-CoV-2 genome sequences collected after June 2022 (triangles; n=226) and random subsamples of the same variants collected around their respective peak prevalence (circles; n=2,195) illustrated on the Time-resolved phylogenetic tree built by IQ-TREE followed by TreeTime.
